# Study of vitamin D receptor gene polymorphisms in a cohort of myocardial infarction patients with coronary artery disease

**DOI:** 10.1186/s12872-021-01959-x

**Published:** 2021-04-16

**Authors:** Damir Raljević, Viktor Peršić, Elitza Markova-Car, Leon Cindrić, Rajko Miškulin, Marta Žuvić, Sandra Kraljević Pavelić

**Affiliations:** 1 Department of Medical Rehabilitation, Medical Faculty, University of Rijeka, B. Branchetta 20, 51000 Rijeka, Croatia; 2 Division of Cardiology, Hospital for Medical Rehabilitation of the Heart and Lung Diseases and Rheumatism “Thalassotherapia Opatija”, M. Tita 188, 51410 Opatija, Croatia; 3 Department of Biotechnology, University of Rijeka, Radmile Matejčić 2, 51000 Rijeka, Croatia; 4 Faculty of Health Studies, University of Rijeka, Viktora Cara Emina 5, 51000 Rijeka, Croatia

**Keywords:** Vitamin D receptor gene polymorphisms, BsmI, Taq1, Myocardial infarction, Coronary artery disease, RT-PCR

## Abstract

**Background:**

Vitamin D deficiency is associated with cardiovascular diseases, including coronary artery diseases (CAD). As vitamin D manifests its biological function through its vitamin D receptor (VDR), VDR gene polymorphisms potentially affect VDR functionality and vitamin D activity. Therefore, the objective of this study was to analyze three well-studied VDR gene polymorphisms—Fok1 (rs2228570), BsmI (rs1544410) and Taq1 (rs731236)—in a cohort of CAD patients after acute myocardial infarction.

**Methods:**

In the presented cross-sectional study, 155 participants with CAD after acute myocardial infarction and 104 participants in a control group without CAD were enrolled. The participants in both groups were Caucasians of European origin. The genotyping of VDR polymorphisms rs2228570, rs1544410 and rs731236 was assessed by RT-PCR.

**Results:**

The results show an association between the T/T genotype of the BsmI (rs1544410) and the G/G genotype of the Taq1 (rs731236) VDR polymorphism and CAD patients after acute myocardial infarction. There was no association between the Fok1 (rs2228570) VDR polymorphism and CAD patients after acute myocardial infarction.

**Conclusion:**

The presented results suggest a potential association of the BsmI (rs1544410) and Taq1 (rs731236) VDR polymorphisms with CAD patients after myocardial infarction.

**Supplementary Information:**

The online version contains supplementary material available at 10.1186/s12872-021-01959-x.

## Background

In recent years, vitamin D levels have been studied as a cardiovascular risk factor by a number of authors [1, 2]. Still, ambiguous data has been obtained on the correlation of the vitamin D levels with some pathologies, such as coronary artery diseases (CAD) [3, 4]. This is not surprising as vitamin D comprises a group of fat-soluble secosterols and their effects should be studied comprehensively along with the corresponding biological mediators and receptors. The vitamin D (calciferol) active metabolite is 1,25-dihydroxyvitamin D (1,25(OH)2D, calcitriol) and its chemical precursors are vitamins D2 (ergocalciferol) and D3 (cholecalciferol). These molecules are involved in important metabolic pathways such as calcium and phosphate homeostasis, bone health and immunological processes. These biological effects are mediated by different biomolecules, in particular the carrier protein—the vitamin D binding protein (VDBP) and the vitamin D receptor (VDR). VDR’s ubiquitous presence in tissues points to the additional roles of vitamin D in the body, including its role in immunity and pathological conditions such as cancer and cardiovascular diseases [5]. In particular, the biological role of vitamin D is tightly related to VDR, a known ubiquitous nuclear receptor. VDR is activated by binding with 1,25(OH)2D and two main mechanisms of VDR activity have been described so far. The first mechanism is through the activation of nuclear VDR and regulation of the transcription of multiple vitamin D-dependent genes through the vitamin D–responsive elements in the genome (VDREs). The second is via the membrane VDR responsible for a rapid response of the cell to an external stimulus [6]. The VDR gene is located on the long arm of chromosome 12 (12 q 12–14) and consists of 11 exons. Polymorphisms within the VDR gene can potentially affect the expression of the VDR gene and mRNA stability, which may eventually impact VDR functionality and vitamin D activity. Over 470 polymorphisms have been identified in the VDR gene so far. However, the most commonly studied polymorphisms are Fok1 (rs2228570), BsmI (rs1544410) and Taq1 (rs731236), named after the restriction enzymes by which they were initially isolated. The VDR gene polymorphisms have been evaluated as independent risk factors in various diseases such as prostate cancer, inflammatory bowel disease, osteopenia and tuberculosis [7], Parkinson's disease [8], diabetes [9] and cardiovascular diseases [10, 11]. However, data on the VDR gene polymorphisms’ association with CAD are inconsistent in the literature. For example, a recent meta-analysis reports on a lack of Fok1, Taq1 and BsmI polymorphisms’ association with CAD [10]. On the other hand, another meta-analysis reports on a protective effect of Fok1 polymorphism in the development of CAD and an increased risk of CAD in subjects with Taq1 polymorphism [12]. Also, a study of both VDBP and VDR polymorphisms in CAD patients showed a strong association between the VDR (rs1544410, G > A) and VDBP (rs7041 T > G) genes polymorphisms and vitamin D deficiency [13], and the latter was also evaluated as a possible risk factor in CAD pathogenesis [14]. In addition, our previously published data show a correlation between the VDBP polymorphism rs4588 and CAD patients with anteroseptal myocardial infarction [15]. Therefore, we expanded our research conducted on VDBP polymorphisms in a CAD cohort with myocardial infarction to the analysis of the Fok1 (rs2228570), BsmI (rs1544410) and Taq1 (rs731236) VDR polymorphisms as well. Our study provides evidence for association between the T/T genotype of the BsmI (rs1544410) and the G/G genotype of the Taq1 (rs731236) VDR polymorphism and CAD patients after acute myocardial infarction. There was no association between the Fok1 (rs2228570) VDR polymorphism and CAD patients after acute myocardial infarction.

## Methods

### Materials and methods

The cross-sectional genotyping study included consecutively recruited 155 subjects with CAD after acute myocardial infarction and consecutively recruited 104 healthy subjects in the control group (Table 1) at the Hospital for Medical Rehabilitation of the Heart and Lung Diseases and Rheumatism "Thalassotherapia-Opatija", Croatia. In the period from 2014 to 2016 a total of 523 patients with myocardial infarction were hospitalized in the “Thalassotherapia Opatija”. Among them, 155 patients that agreed to sign the informed consent, were in compliance with the inclusion criteria of the presented study. The subjects were enrolled in the late winter of 2014 and during autumn, winter and spring months of 2015 and 2016, to avoid effects of the strong insulation on the vitamin D levels during the summer. All subjects in the CAD and the control group were Caucasians of European descent and residents of the Primorje-Gorski kotar County (Republic of Croatia). The control group comprised significantly more women than the CAD group (*p* = 0.002). There was no age difference between groups by gender (t-test, *p* = 0.547). Eligibility criteria: for the CAD group, patients older than 18 of both genders with proven CAD who had gone through the program of cardiac rehabilitation after an acute myocardial infarction as well as percutaneous coronary revascularization (PCI); for the control group: healthy patients older than 18 of both genders without a previous history of CAD who had been submitted to a routine check-up at “Thalassotherapia Opatija”.

**Table 1:** Age, body mass index (BMI) (t-test), blood pressure and other prevalence risk factors distribution between coronary artery disease (CAD) and control group (chi square analysis. Fisher exact test was done for COPD)

	All (n = 259)	CAD (n = 155)	Control (n = 104)	*p (t-test)*
Age/ys. mean. ± SD	53 ± 12	58 ± 11	47 ± 11	< 0.001
BMI/kgm^−2^. mean ± SD	28.0 ± 4,3	28.5 ± 4,0	27.2 ± 4,5	0.017
RR (S)/mmHg. mean ± SD	124 ± 15	121 ± 15	127 ± 16	0.005
RR (D)/mmHg. mean ± SD	78 ± 10	78 ± 10	78 ± 9	0.887

				*p (χ*^*2*^* test)*
Gender/female N (%)	64 (24.7)	28 (18,1)	36 (34,6)	0.002
Arterial hypertension N (%)	157 (60.6)	117 (75.5)	40 (38.5)	< 0.001
Hyperlipoproteinemia N (%)	200 (77.2)	122 (78.7)	78 (75.0)	0.485
Diabetes Mellitus N (%)	38 (14.7)	29 (18.7)	9 (8.7)	0.025
COPD N (%)	5 (1.93)	5 (3.2)	0 (0)	0.085*
Smoking N (%)	108 (41.7)	86 (55.5)	22 (21.2)	< 0.001
Positive family history N (%)	154 (59.5)	93 (60.0)	61 (58.7)	0.829

#### Exclusion criteria

Proven malignancy, known primary hyperparathyroidism, calcium and vitamin D replacement therapy and patients with renal failure stage G4 and above according to the KDIGO 2012 chronic kidney disease classification, corresponding to eGFR lower than 30 mL/min/1,73m^2^. The sampling was performed during cardiac rehabilitation at least 10 days after the acute event. Data on chronic drug treatment of the tested cohort is presented in the Supplement file (Additional file 1: Table S13). The study was approved by the Ethics Committee of “Thalassotherapia Opatija” and the Ethics Committee of the Medical School in Rijeka. Data on age, gender, medical history, habits and risk factors (smoking, arterial hypertension, diabetes and family history) in the CAD and the control group were taken (Table 1). The type and localization of acute myocardial infarction was determined by electrocardiography (myocardial infarction with or without ST elevation) and coronary angiography. All subjects signed a written informed consent that was in accordance with the Helsinki Declaration and approved by the Ethics Committee of “Thalassotherapia Opatija” and the Ethics Committee of the Medical School in Rijeka. The detailed procedures used in the clinical evaluation of patients are explained in the Supplement file (Additional file 1: Subjects and procedures used for clinical evaluation of patients). Part of the results from this study involving the analysis of VDBP and serum vitamin D levels were previously published [15].

### DNA extraction and genetic analysis

The genomic DNA was extracted from 200 μL of peripheral blood using the QIAamp DNA Blood Mini Kit (Qiagen, Hilden, Germany) on a QIAcube robotic workstation (Qiagen), as per manufacturer's instructions. DNA quality and concentration were measured using a BioDrop spectrophotometer. The genotyping of VDR gene polymorphisms rs2228570, rs1544410 and rs731236 was performed with TaqMan Pre-designed SNP Genotyping Assay on an Applied Biosystems 7500 fast real-time PCR device as per manufacturer's instructions. The PCR conditions of the cycles were set at 95 °C for 10 min, followed by 45 cycles of denaturation at 95 °C for 15 s and annealing/extension at 60 °C for 1 min. The obtained results were analyzed with allelic discrimination analysis software present on the Applied Biosystems 7500 fast PCR instrument.

### Vitamin D serum level measurement

After overnight fasting serum samples were collected for 118 patients with CAD and 70 control subjects and stored at − 80 °C. The levels of 25 (OH) D2 and 25 (OH) D3 were measured using ACQUITY UPLC I-Class and Xevo TQD (Milford, Massachusetts, USA). MassLynx software (Waters, USA) was used to create the experiment and analyze the results, while the UPLC parameters were controlled using the ACQUITY UPLC console. The solid phase extraction procedure and liquid chromatography, tandem mass spectrometry (LC–MS/MS) are described in the Supplement file (Additional file 1: Solid phase extraction procedure and LCMS/MS and Table S2). The results of vitamin D serum levels measurements for the study cohort have been published already [15].

### Statistics

To estimate the expected genotype distributions in the CAD group and the control group, the Hardy–Weinberg equation of equilibrium was used. The collected data were processed by the Dell Statistica statistical software, Version 12, Dell Inc. (2015). The categorical variables are presented in frequencies and percentages, and a comparison is made by using the Person chi-square (*X*2) test and the Bonferroni correction for multiple testing is applied where suitable. The association of categorical variables is expressed by OR with 95% CI. The continuous variables of normal distribution were tested by the Kolmogorov–Smirnov test and presented as mean ± standard deviation. A t-test was used to compare values between the two groups, while for the multiple groups the comparison was performed by variance analysis, the ANOVA test and the post-hoc Sheaffe test. Statistical significance was set at 0.05.

## Results

The tested cohort of patients comprised 259 subjects, where 155 (59.8%) were patients diagnosed with coronary artery disease after myocardial infarction (CAD patients) and 104 (40.1%) were controls. Considering the allele frequency in the general population and the hypothetical allele frequency in CAD with a Type I error rate of 0.05 and an analysis power of 80%, the minimum sample size for Fok1 (rs2228570), BsmI (rs1544410), and Taq1 (rs731236) was set as 144, 51 and 116 subjects, respectively. In the CAD group, 124 subjects (80%) had ST elevation myocardial infarction (STEMI) while 31 subjects (20%) had non ST elevation myocardial infarction (NSTEMI). The most common localization of myocardial infarction was the anteroseptal wall of the left ventricle (61 subjects, 39.4%) and the inferior posterior wall of the left ventricle (61 subjects, 39.4%), while only 5 subjects (3.2%) had a lateral myocardial infarction. The subjects within the CAD group had a previous diagnosis of arterial hypertension and were receiving antihypertensive therapy (Table 1). Therefore, lower blood pressure values in the CAD group were attributed to the adequate antihypertensive therapy. The value of blood pressure was under the cutoff for arterial hypertension (AH) in both groups; hence, the difference was not considered relevant for this study. Analysis of the individual VDR polymorphism alleles showed no statistically significant differences in frequency between the CAD and the control group (Additional file 1: Table S3). Genotype analysis showed no statistically significant differences in Fok1 VDR rs2228570 genotype frequency between the CAD and the control group. The C/T genotype of BsmI VDR rs1544410 was significantly less present in the CAD group compared to the control group (*χ*2 test, *p* = 0.006), while in the Taq1 VDR rs731236 analysis, the A/G genotype was significantly less frequent in the CAD group compared to the control group (*χ*2 test, *p* = 0.01) (Table 2). When adding gender as a factor to the presented data, the results of log-linear frequency analyses of the tables showed no significant interaction (correction of p-level by 0.003). Since there is no observed age difference by gender, the age can be assumed as not comprising to the overall effect. Analysis of the inheritance models of the BsmI VDR polymorphism showed a protective role of the T/C genotype under codominant (C/C vs T/C: OR 1.82; 95% CI 1.07–3.08, *p* = 0.027) and overdominant (C/C + T/T vs T/C: OR = 2.08; 95% CI 1.25–3.44, *p* = 0.004) genetic models in CAD patients. In addition, under the recessive model (C/C + T/C vs T/T: OR = 0.35; 95% CI 0.41–0.90, *p* = 0.023) the T/T genotype carriers are more susceptible to CAD (Table 3). Furthermore, inheritance model analysis of the Taq1 polymorphism showed that under the recessive model (A/A + A/G vs G/G: OR = 0.31; 95% CI 0.11–0.83, *p* = 0.016) the G/G genotype is associated with a significantly higher risk for CAD, and the A/G genotype under the overdominant model (A/A + G/G vs A/G: OR = 1.86; 95% CI 1.12–3.08, *p* = 0.016) plays a protective role (Table 4).

**Table 2:** Genotype frequencies of vitamin d polymorphisms VDR (rs2228570, rs1544410, rs731236) in coronary artery disease CAD group in comparison with control

	CAD		Control		*P (χ*^*2*^* test)*
	*N*	%	*N*	%	
VDR (rs2228570)					
A/A	21	13.5	18	17.3	0.67
A/G	84	54.2	52	50.0	
G/G	50	32.3	34	32.7	
Total	155	100.0	104	100.0	
VDR (1544410)					
C/C	72	46.5	39	37.5	0.006
C/T	60	38.7	59	56.7	
T/T	23	14.8	6	5.8	
Total	155	100.0	104	100.0	
VDR (rs731236)					
A/A	76	49.0	45	43.3	0.01
A/G	57	36.8	54	51.9	
G/G	22	14.2	5	4.8	
Total	155	100.0	104	100.0	

Bonferroni corrected alpha level = 0.017

**Table 3:** Inheritance models for BsmI VDR (rs1544410) polymorphism in CAD and control groups

VDR (1544410) model		CAD	Control	*p*	OR	− 95% CL	+ 95% CL
Codominant Model 1 C/C vs T/C	C/C	72	39	0.027	1.82	1.07	3.08
	T/C	60	59				
Codominant Model 2 C/C vs T/T	C/C	72	39	0.138	0.48	0.18	1.28
	T/T	23	6				
Dominant Model C/C vs T/C + T/T	C/C	72	39	0.154	1.45	0.87	2.40
	T/C + T/T	83	65				
Recessive Model C/C + T/C vs T/T	C/C + T/C	132	98	0.023	0.35	0.14	0.90
	T/T	23	6				
Overdominant MoDEL C/C + T/T vs T/C	C/C + T/T	95	45	0.004	2.08	1.25	3.44
	T/C	60	59				
Additive Model C/C vs 2 T/T + T/C	C/C	72	39	0.397	1.24	0.76	2.02
	2 T/T + T/C	106	71				

Bonferroni corrected alpha level = 0.008

**Table 4:** Inheritance models for Taq1 VDR (rs731236) polymorphism in CAD and control groups

VDR (rs731236) Model		CAD	Control	*p*	OR	− 95% CL	+ 95% CL
Codominant Model 1 A/A vs A/G	A/A	76	45	0.078	1.6	0.95	2.70
	A/G	57	54				
Codominant Model 2 A/A vs G/G	A/A	76	45	0.064	0.38	0.14	1.08
	G/G	22	5				
Dominant Model A/A vs A/G + G/G	A/A	76	45	0.362	1.26	0.77	2.08
	A/G + G/G	79	59				
Recessive Model A/A + A/G vs G/G	A/A + A/G	133	99	0.016	0.31	0.11	0.83
	G/G	22	5				
Overdominant ModEL A/A + G/G vs A/G	A/A + G/G	98	50	0.016	1.86	1.12	3.08
	A/G	57	54				
Additive Model A/A vs 2G/G + A/G	A/A	76	45	0.783	1.07	0.66	1.74
	2G/G + A/G	101	64				

Bonferroni corrected alpha level 0.008

The genotype frequencies comparison for the STEMI CAD group vs. control showed no statistically significant differences for the Fok1 (rs2228570) polymorphism, whereas for the BsmI (rs1544410) VDR the C/T genotype frequency was significantly lower and the T/T genotype frequency was significantly higher in the STEMI CAD group (*χ*^*2*^ test, *p* = 0.017) (Additional file 1: Table S4). By analyzing the inheritance model of the BsmI polymorphism, under the recessive model the T/T genotype was associated with a 2.94-fold higher risk for STEMI CAD (95% CI 0.13–0.88, *p* = 0.022) (Additional file 1: Table S5). For the Taq1 (rs731236) polymorphism, the frequency of the A/G genotype was significantly lower and the G/G genotype was significantly higher in STEMI CAD compared to the control group (*χ*^*2*^ test, *p* = 0.020) (Additional file 1: Table S4). Under the recessive inheritance model, the Taq1 G/G genotype was associated with a 3.33-fold higher risk of STEMI CAD (95% CI 0.11–0.83, *p* = 0.016) (Additional file 1: Table S6). Furthermore, as with STEMI CAD, there is an association of the BsmI polymorphism with the NSTEMI CAD group (*χ*^*2*^ test, *p* = 0.022), and the Taq1 polymorphism with the NSTEMI CAD group (*χ*^*2*^ test, *p* = 0.046) relative to the control group (Additional file 1: Table S7).

Considering that smoking is a significant risk factor for the development of myocardial infarction in CAD patients, we analyzed the group of non-smokers in the CAD group in comparison with the non-smokers in the control group, and a statistical difference in genotype frequency of the BsmI polymorphism between the CAD and the control group was observed (*χ*^*2*^ test, *p* = 0.039) (Additional file 1: Table S8). No differences were observed in tested polymorphisms’ genotype frequency between the CAD smokers and the smokers in the control group (Tables 5, 6), as well as between the smokers and non-smokers generally (Additional file 1: Table S9). These results may suggest that the examined VDR polymorphism genotypes influence CAD development independently of smoking. An analysis of the study groups according to gender showed a marginally significant difference between the CAD and the control group for the BsmI and Taq1 VDR polymorphism genotype frequency in males (*χ*^*2*^ test, *p* = 0.058 and *χ*^*2*^ test, *p* = 0.063, respectively (Additional file 1: Table S10). In females, there was a marginally significant difference in the BsmI polymorphism (*χ*^*2*^ test, *p* = 0.070) and a significant difference in the Taq1 polymorphism (*χ*^*2*^ test, *p* = 0.050) between the CAD group and the control group (Additional file 1: Table S11). Interestingly, no statistically significant differences were found for the BsmI and Taq1 polymorphisms in a comparison of the VDR polymorphisms’ genotype frequencies by gender, while in the Fok1 polymorphisms the A/G genotype was significantly more frequent in males than females (*χ*^*2*^ test, *p* = 0.006) (Additional file 1: Table S12).

**Table 5:** Analysis of 25 hydroxyvitamin D2 (25(OH)D2), 25 hydroxyvitamin D3 (25(OH)D3) and total vitamin D serum levels for different VDR (rs2228570, rs1544410, rs731236) genotypes

	N	25 hydroxyvit D2 /nmol/L	25 hydroxyvit D3 /nmol/L	Total vit D /nmol/L
VDR (rs2228570)				
A/A	31	3.08 ± 3.90	57.71 ± 24.52	60.79 ± 24.65
A/G	93	2.12 ± 3.33	59.38 ± 31.65	61.50 ± 31.39
G/G	64	2.61 ± 3.63	52.06 ± 22.46	54.67 ± 22.77
* p (ANOVA)*	188	0.385	0.261	0.294
* p* (factorial ANOVA, by gender)		0.859	0.580	0.619
VDR (1544410)				
C/C	74	2.78 ± 3.64	55.91 ± 25.22	58.69 ± 25.21
C/T	90	2.26 ± 3.48	55.67 ± 28.77	57.93 ± 28.46
T/T	24	2.11 ± 3.48	62.36 ± 31.64	64.46 ± 32.39
* p (ANOVA)*	188	0.573	0.557	0.586
* p (factorial ANOVA, by gender)*		0.743	0.782	0.795
VDR (rs731236)				
A/A	82	2.89 ± 3.70	55.79 ± 25.38	58.69 ± 25.32
A/G	83	2.18 ± 3.44	55.66 ± 28.80	57.84 ± 28.48
G/G	23	1.79 ± 3.19	62.99 ± 32.20	64.78 ± 33.08
* p (ANOVA)*	188	0.276	0.503	0.563
* p (factorial ANOVA, by gender)*		0.847	0.862	0.878

When adding gender in Factorial ANOVA analyses, no interaction of gender*VDR has been noticed (p > 0.05 in all analyses, as presented). Since there was no observed age difference by gender, the age can be assumed as not comprising to the overall effect

Results are presented as mean values in nmol/L ± standard deviation (SD)

**Table 6:** Contingency table for VDR polymorphisms (rs2228570, rs1544410, rs731236) regarding smoking

	CAD (smoking)		Control (smoking)		*p* (χ^2^ test)
	*N*	%	*N*	%	
VDR (rs2228570)					
A/A	10	11.6	3	13.6	0.84
A/G	47	54.7	13	59.1	
G/G	29	33.7	6	27.3	
Total	86	100.0	22	100.0	
VDR (1,544,410)					
C/C	41	47.7	9	40.9	0.104
C/T	34	39.5	13	59.1	
T/T	11	12.8	0	0.0	
Total	86	100.0	22	100.0	
VDR (rs731236)					
A/A	44	51.2	10	45.5	0.109
A/G	31	36.0	12	54.5	
G/G	11	12.8	0	0.0	
Total	86	100.0	22	100.0	

When adding gender as a factor to the analyses presented, the results of log-linear frequency analyses of the tables showed no significant interaction (correction of p-level by 0.002)

Bonferroni corrected alpha level = 0.017

## Discussion

Vitamin D has a significant role in the normal functioning of the body. Through its VDR receptor, vitamin D participates in the expression of approximately 3% of the human genome [16] and directly or indirectly participates in the regulation of cellular functions, such as cellular growth, DNA repair, cell differentiation, apoptosis, membrane transport, cell metabolism, cell adherence and oxidative stress [17]. The influence of vitamin D on calcium metabolism is well-known; however, in recent years there has been a growing awareness of the extracellular role of vitamin D. Serum vitamin D levels affect fibrinolysis, lipid profile, thrombogenicity, growth and regeneration of the endothelial and smooth muscle cells [18] and they are associated with arterial hypertension and the metabolic syndrome [19]. We have previously shown that the serum levels of 25(OH)D, considered clinically relevant for the assessment of vitamin D levels in the organism [20], were normal in the CAD group evaluated in the presented study as well [15]. However, the VDBP (rs4588) genotype’s G/G presence correlated with the higher levels of 25(OH)D in the serum show a relationship of the VDBP genetic status and vitamin D levels in the serum [15]. Also, no correlation of serum vitamin D levels with the frequencies of the specifically investigated VDR genotypes has been found in our study (Table 6), which may suggest the independence of the VDR genotype variants and serum vitamin D levels on CAD. Polymorphisms in the VDR gene, can also potentially affect VDR expression and the stability of VDR mRNA, which may eventually affect VDR functionality and vitamin D activity [7]. Analysis of the Fok1 (rs2228570) VDR polymorphism did not reveal a significant difference in genotype frequency between the two studied groups. These results are consistent with a study by Pan et al. in a Chinese population with no finding of association between Fok1 polymorphism and CAD [21], and with a meta-analysis by Iranian scientists with no indication of association between Fok1 polymorphism and CAD [10]. On the other hand, our results do not match those of Nezhad et al., who found an association of Fok1 polymorphism with serum vitamin D values. They also found Fok1 association with the degree of coronary artery collateralization in CAD, a parameter not assessed in our study [22]. He et al. found a protective effect of the Fok1 polymorphism G/G genotype in CAD in a Chinese population [23]. Finally, a meta-analysis of Chinese authors found a protective effect of the G allele Fok1 polymorphism on the development of coronary disease [12]. Moreover, our data provide evidence on association between the T/T genotype BsmI (rs1544410) VDR and the G/G genotype Taq1 (rs731236) with CAD in the studied cohort. In the literature, however, data on the impact of these VDR gene polymorphisms in CAD are inconsistent. Pan et al. do not find an association of BsmI VDR polymorphism with CAD in the Chinese population [21]. In the Dutch population, Van Schooten et al. recognize BsmI as a marker in assessing susceptibility to CAD [24]. Ortlepp et al. found an association of BsmI polymorphism with coronary disease in the German population with type II diabetes [25]. However, in a study performed on a larger population, no association of BsmI polymorphism and the prevalence and severity of CAD has been established [26]. In that study, there was no comparison with the control group, and the distribution of alleles was not in accordance with the Hardy–Weinberg equation of equilibrium. A group of French authors in two independent cohorts found an association of BsmI minor alleles and Taq1 minor alleles with an increased risk of CAD in a population with type II diabetes [27]. Two meta-analyses were conducted to investigate VDR polymorphisms in CAD, however but the results are also inconsistent. A group of Iranian scientists conducted a meta-analysis that did not indicate an association between Taq1 and BsmI polymorphisms and CAD [10]. On the other hand, a meta-analysis by a group of Chinese authors found an increased risk for CAD forTaq1 polymorphism and BsmI polymorphism in Caucasians [12]. The inconsistency of the results in the studies may be due to the heterogeneity of the populations and the severity of coronary disease. It means that it might be helpful to define CAD parameters in more detail in genetic traits research that can then be associated with exact CAD patient groups. For example, most studies define CAD as > 50% stenosis of at least one coronary artery as assessed by coronary angiography. A more objective assessment of the significance of the degree of stenosis should be done additionally by any modern method, such as intravascular ultrasound (IVUS), instant wave-free ratio (IFR), fractional flow reserve (FFR) or optical coherence tomography (OCT) to minimize false positive findings.

In our study, a difference was observed between the study groups in the history of previously diagnosed arterial hypertension. Blood pressure was lower in the CAD group, probably due to optimal medical treatment. However, since the measured blood pressure values were normal in both study groups, we do not consider this difference to be of relevance for the results of our study. Also, there is a significant difference between the tested groups regarding smoking (Table 1). As smoking is recognized as a major risk factor for the development of CAD, this difference may be a confounding factor. Further analyses of subpopulations of smokers in the CAD and control groups suggest however, that the frequencies of certain genotypes for VDR are independent risk factors for the development of CAD regarding smoking.

### Study limitations

The main limitation of the presented study may be in a rather small size of the samples. However, the power calculated according to the allele frequency in the general population, the hypothetical frequency of CAD with the error level type I at 0.05 and the power of analysis at 80% showed the minimum sample size for Fok1 (rs2228570), BsmI (rs1544410), and Taq1 (rs731236) to be 144, 51 and 116 subjects, respectively. The observed differences in gender, AH and smoking may add bias to the results as well.

## Conclusions

In brief, the results suggest that the T/T genotype of the BsmI (rs1544410) VDR polymorphism and the G/G genotype of the Taq1 (rs731236) VDR polymorphism figured more prominently among the myocardial infarction CAD patients. Nevertheless, a larger, well-designed prospective study involving different populations and disease stages might be considered to further determine the association of a specific VDR genotype with CAD, especially in view of the rather small sample size of the studied cohort. Even though the sample size of the cohort is based on a calculated sample power, a larger cohort might provide additional information about the studied VDR polymorphisms in a larger Croatian population cohort. Lastly, the observed differences in smoking and gender can also be additionally evaluated as potential confounding factors.

## Supplementary Information

**Additional file 1.** Additional data for the paper: Observational study of vitamin D receptor gene polymorphisms in a cohort of myocardial infarction patients with coronary artery disease.

## Data Availability

The datasets generated and/or analyzed during the current study are available in the Figshare repository (link to the dataset: https://figshare.com/s/935cb8e968c9e0cb5366).

## Abbreviations

CAD: Coronary artery diseases
VDR: Vitamin D receptor
VDBP: Vitamin D binding protein
VDREs: Vitamin D–responsive elements
PCI: Percutaneous coronary revascularization
LC–MS/MS: Liquid chromatography, tandem mass spectrometry
STEMI: ST elevation myocardial infarction
NSTEMI: Non ST elevation myocardial infarction
AH: Arterial hypertension
IVUS: Intravascular ultrasound
IFR: Instant wave-free ratio
FFR: Fractional flow reserve
OCT: Optical coherence tomography
