# Supplementary material for: Study of vitamin D receptor gene polymorphisms in a cohort of myocardial infarction patients with coronary artery disease
Source: BMC Cardiovasc Disord. 2021 Apr 16;21:188. doi: 10.1186/s12872-021-01959-x (PMC8052753; doi:10.1186/s12872-021-01959-x)
Supplement: Supplementary file 1 — Additional file 1. Additional data for the paper: Observational study of vitamin D receptor gene polymorphisms in a cohort of myocardial infarction patients with coronary artery disease. [file 12872_2021_1959_MOESM1_ESM.docx]

**Additional data for the paper:**

**Observational study of vitamin D receptor gene polymorphisms in a cohort of myocardial infarction patients with coronary artery disease**

**Damir Raljević^1±^, Viktor Peršić^1*^, Elitza Markova-Car^2±^, Leon Cindrić^2^, Rajko Miškulin^1^, Marta Žuvić^2^, Sandra Kraljević Pavelić^3*^**

^1^ Department of Medical Rehabilitation, Medical Faculty, University of Rijeka, B. Branchetta 20, 51000 Rijeka, Croatia; Division of Cardiology, Hospital for Medical Rehabilitation of the Heart and Lung Diseases and Rheumatism "Thalassotherapia-Opatija", M. Tita 188, 51410 Opatija, Croatia

^2^ University of Rijeka, Department of biotechnology, Radmile Matejčić 2, 51000 Rijeka, Croatia

^3^ University of Rijeka, Faculty of Health Studies, Viktora Cara Emina 5, Rijeka, 51000, Croatia

**± Equal contribution**

*** Correspondence:**

**Sandra Kraljević Pavelić**, University of Rijeka, Faculty of Health Studies, Viktora Cara Emina 5, Rijeka, 51000, Croatia, [sandrakp@uniri.hr](mailto:sandrakp@uniri.hr)

**For the clinical part: Viktor Peršić**, Department of Medical Rehabilitation, Medical Faculty, University of Rijeka, B. Branchetta 20, 51000 Rijeka, Croatia; Division of Cardiology, Hospital for Medical Rehabilitation of the Heart and Lung Diseases and Rheumatism "Thalassotherapia-Opatija", M. Tita 188, 51410 Opatija, Croatia, [viktor.persic@ri.t-com.hr](mailto:viktor.persic@ri.t-com.hr)

**Content**

**[1.](#_Toc55469236)****[Subjects and procedures used for clinical evaluation of patients.](#_Toc55469236)** [3](#_Toc55469236)

**[2.](#_Toc55469237)****[Clinical parameters in the tested cohort](#_Toc55469237)** [6](#_Toc55469237)

**[3.](#_Toc55469238)****[Solid phase extraction procedure and LC-MS/MS](#_Toc55469238)** [7](#_Toc55469238)

**[4.](#_Toc55469239)****[Additional tables](#_Toc55469239)** [9](#_Toc55469239)

# **Subjects and procedures used for clinical evaluation of patients.**

A total of 124 patients (80%) had acute myocardial infarction with the ST segment elevation (STEMI), while 31 patients (20%) had acute myocardial infarction without ST segment elevation (NSTEMI). In the CAD group 94 (60.6%) of the data subjects had been subjected to percutaneous coronary intervention (PCI) on the anterior descending branch of the left coronary artery. PCI on the circumflexed branch of the left coronary artery was performed in 25 data subjects (16.1%), while in 43 data subjects (27.7%) it was performed on the right coronary artery. In the CAD group 117 patients (75.5%) of the CAD group have suffered from arterial hypertension, 122 CAD subjects (78.7%) have suffered from hypolipoproteinemia, 29 CAD subjects (18.7%) have suffered from diabetes, 5 CAD subjects (3.2%) have suffered from chronic obstructive pulmonary disease (COPD). A total number of 86 data subjects (55.5%) were smokers and 93 CAD subjects (61%) had a positive family history of cardiovascular diseases.

A statistically significant difference between CAD and control group was observed in gender distribution (81.9% male in CAD group *vs.* 65.4% male in control group). Statistically significant difference was also observed between CAD and control group in age distribution (58 years *vs.* 47 years, p<0.001). There was also a difference in the body mass index (28.5 *vs.* 27.2 kg/m^2^, p=0.017) without clinical significance as both values were in the overweight range. While predisposing factors including hypolipoproteinemia, COPD prevalence and positive family history of cardiovascular diseases showed no differences between groups, statistically significant difference between groups was observed in prevalence of arterial hypertension (AH) (75.5% *vs.* 38.5%, p < 0.01), diabetes mellitus (18.7 *vs.* 8.7, p=0.025) and smoking (55.5% *vs.* 21.2%, p<0.001) (Table S1)

Blood samples of the affected subjects were taken between 7 am and 8 am, the second day after the admission to rehabilitation treatment. Serum levels of vitamin D (25 (OH) D) were determined by the mass spectrometry (Xevo TQ-S, Waters corporation, Milford USA), natriuretic peptide (BNP), high sensitive C-reactive protein (HsCRP) and other routine laboratory findings (complete blood count – CBC, plasma glucose levels, urea, creatinine, sodium, potassium, calcium, AST, ALT, GGT, ALP; bilirubin, cholesterol, triglycerides, HDL, LDL TSH. Erythrocyte sedimentation was defined by Westergreen method (Becton Dickinson, Franklin Lakes, NJ, USA.). Hemoglobin was defined via “Sulfolyser oxidizing method“, erythrocytes and hematocrit via “Hydro Dynamic Focusing method“ on hematological analyzer (Hematology analyzer XS-1000i, Sysmex, Kobe, Japan). Leukocytes, neutrophils, eosinophils, basophils, lymphocytes and monocytes were determined by the flow cytometry with the help of/ means semiconductor laser on a hematologic analyzer. (Hematology analyzer XS-1000i, Sysmex, Kobe, Japan). MCV, MCH and MCHC were calculated on the bases of the data obtained on the hematological analyzer. Bilirubin, AST, ALT, GGT, Na +, K +, urea, creatinine, hsCRP (in English: high sensitivity C- reactive protein,) cholesterol, triglycerides, HDL, LDL, TSH and urates were determined by photometric method using Dimension Xpand, (Siemens Healthcare Diagnostics, Newark (DE) USA). All the laboratory parameters were determined in the laboratory of Specialized hospital for cardiac, pulmonary and rheumatic diseases “Thalassotherapia Opatija”. During this research, the following anthropometric measurements were taken (all by using the same devices): height in meters (m), weight in kilograms (kg) by using manual scale. Systolic Blood Pressure (SBP) and Diastolic Blood Pressure (DBP) were measured in persons while sitting, after taking a rest for at least 15 minutes. BMI is calculated as weight (kg) / height (m^2^).

# **Clinical parameters in the tested cohort**

Table S1. Hematological and biochemical parameters of the tested cohort. Test results were compered between the control group and CAD. Statistically relevant differences at p<0.05 are denoted with an asterisk (*).

|  | **CAD** | | **Control** | | *p* |
| --- | --- | --- | --- | --- | --- |
|  | N | *value* | N | *value* |  |
| **ES** /mm*. median (IQR)* | 153 | 26 (9-34) | 101 | 10 (5-12) | <0.001 |
| **Erythrocytes** /10^12^/L. *mean.± SD* | 153 | 4.73 ± 0.49 | 101 | 4.90 ± 0.43 | 0.004 |
| **Hb** /g/L. *mean.± SD* | 154 | 146 ± 14 | 101 | 150 ± 15 | 0.04 |
| **Hct**. *mean.± SD* | 154 | 0.43 ± 0.04 | 101 | 0.44 ± 0.04 | 0.132 |
| **MCV** / fL*. median (IQR)* | 154 | 91 (88-94) | 101 | 90 (86-92) | 0.006 |
| **MCH** / pg*. median (IQR)* | 154 | 31 (30-32) | 101 | 31 (30-32) | 0.085 |
| **MCHC** / g/L*. median (IQR)* | 154 | 339 (334-345) | 101 | 341 (335-347) | 0.225 |
| **RDW** / fL*. median (IQR)* | 147 | 42 (40-44) | 101 | 41 (40-43) | 0.025 |
| **Leukocytes** /10^9^/L. *mean.± SD* | 154 | 8.2 ± 2.1 | 101 | 6.8 ± 1.9 | <0.001 |
| **NEU** / %. *mean.± SD* | 154 | 57.0 ± 8.2 | 101 | 54.2 ± 8.3 | 0.008 |
| **LY** / %. *mean.± SD* | 154 | 29.0 ± 7.8 | 101 | 32.4 ± 7.5 | <0.001 |
| **MO** / %. *mean.± SD* | 154 | 10.5 ± 3.0 | 101 | 9.9 ± 2.4 | 0.135 |
| **EO** / %. *mean.± SD* | 154 | 3.0 ± 1.7 | 101 | 3.0 ± 1.7 | 0.809 |
| **BA** / %. *mean.± SD* | 154 | 0.4 ± 0.2 | 101 | 0.4 ± 0.2 | 0.249 |
| **TRC** /10^9^/L. *mean.± SD* | 154 | 265 ± 73 | 101 | 255 ± 55 | 0.240 |
| **Gluc /** mmol/L. *median (IQR)* | 154 | 6.2 (5.1-6.7) | 101 | 5.4 (5.0-5.9) | <0.001 |
| **Urea /** mmol/L*. median (IQR)* | 154 | 5.8 (4.8-7.0) | 101 | 5.3 (4.4-6.1) | <0.001 |
| **Bil T /** μmol/L*. median (IQR)* | 154 | 12 (9-15) | 101 | 10 (8-13) | 0.135 |
| **Bil D /**μmol/L*. median (IQR)* | 153 | 3 (2-3) | 100 | 2 (1-3) | <0.001 |
| **Urates /** rates /*median (IQR)* | 153 | 324 (284-380) | 100 | 307 (237-356) | 0.012 |
| **Creatinine /**L*.median(IQR)* | 153 | 88 (80-99) | 100 | 82 (73-93) | <0.001 |
| **eGFR /**ml/min/1.73m^2^. *mean.±SD* | 153 | 75 ± 17 | 100 | 83 ± 15 | <0.001 |
| **Tgl /**mmol/L. *. median (IQR)* | 154 | 1.2 (0.9-1.8) | 101 | 1.2 (0.8-1.6) | 0.918 |
| **Cholesterol/**mmol/L. *median (IQR)* | 154 | 3.8 (3.2-4.5) | 101 | 5.7 (5.0-6.5) | <0.001 |
| **HDL /**mmol/L. *median (IQR)* | 153 | 1.1 (0.9-1.4) | 101 | 1.5 (1.2-1.8) | <0.001 |
| **LDL /**mmol/L. *median (IQR)* | 154 | 2.0 (1.6-2.7) | 100 | 3.4 (2.9-4.0) | <0.001 |
| **AST /**U/L*. median (IQR)* | 152 | 27 (22-36) | 101 | 22 (18-28) | <0.001 |
| **ALT /**U/L*. median (IQR)* | 154 | 43 (35-58) | 101 | 37 (29-48) | 0.041 |
| **GGT /**U/L*. median (IQR)* | 154 | 34 (28-52) | 102 | 28 (22-41) | 0.019 |
| **ALP /**U/L. *median (IQR)* | 151 | 74 (60-91) | 7 | 65 (48-105) | 0.500 |
| **K^+^ /** mmol/L. *median (IQR)* | 154 | 4.6 (4.3-4.8) | 99 | 4.3 (4.0-4.6) | <0.001 |
| **Na^+^ /** mmol/L*. median (IQR)* | 154 | 141 (140-142) | 100 | 141 (140-142) | 0.397 |
| **HsCRP /** mg/L*. median (IQR)* | 110 | 3.1 (1.3-10.7) | 99 | 1.5 (0.9-2.9) | <0.001 |
| **TSH /**mlU/L*. median (IQR)* | 108 | 1.8 (1.0-2.7) | 101 | 1.9 (1.3-2.5) | 0.479 |

# **Solid phase extraction procedure and LC-MS/MS**

For solid phase extraction (SPE), methanol and water (Honeywell, Morris Plains, New Jersey, USA) were used for preparation of working solutions as follows: 80% methanol/20% isopropyl alcohol (IPA) (v/v) (Sigma-Aldrich, St. Louis, Missouri, USA), 60% methanol (aq.), 5% methanol (aq.), 95% methanol /5% IPA (v/v). Zinc sulfate (0.2 M), 2 mM ammonium acetate/0.1% formic acid (v/v) (aq.), 2 mM ammonium acetate/0.1% formic acid (v/v) in methanol were purchased from (Sigma-Aldrich, St. Louis, Missouri, USA). Briefly, for SPE 20 µL of internal standard mix was added into 150 µL plasma samples aliquots and vortexed briefly. 150 µL of zinc sulphate (0.2 M) was added to the samples followed by addition of 600 µL of 100% methanol. Samples were then centrifuged 10 minutes at 14 000 rpm. After SPE, supernatant containing 25(OH) vitamin D2 and D3 was obtained and collected for each sample. Samples were additionally eluted by use of OASIS® HLB µElution plate 30µm (Waters, Milford, Massachusetts, USA) for the final protein clean-up. Calibrator mix 25-OH Vitamin D3 and D2 solutions (Sigma-Aldrich, St. Louis, Missouri USA) of known concentrations were used to assess the linearity of the measurement. Quality control (QC) mix of 25-OH vitamin D3 and D2 solutions ClinChek® (LGC, Teddington, UK) were used as measurement quality assurance. Internal standard (250 ng/mL) was the referent solution of vitamin D2 (triply deuterated) and D3 (doubly deuterated) (Waters, Milford, Massachusetts, USA), these were added to each sample, QCs and calibrators, relative to which all 25-OH vitamin D2 and D3 and concentrations were measured (internal control is to account for possible sample loss during preparation). UPLC settings included BEH-phenyl column (2.1 x 50 mm, 1.7 µm) (Waters, Milford, Massachusetts, USA), used to separate the OH vitamins D from lipids, mobile phase A containing 2 mM ammonium acetate in 0.1% formic acid (aq.), mobile phase B consisted of 2 mM ammonium acetate in 0.1% formic acid (in methanol), gradient elution (0.45 mL/min flow, 4.2 min run time, 10 µL injection volume, 45°C column temperature, 10 °C sample temperature, weak wash solution containing 60%/40% (v/v) methanol/water and strong wash solution containing 100% methanol). The mass spectrometer settings were set on electrospray ionization positive mode, 1.5 kV capillary voltage, 450°C desolvation temperature, 1100 L/h desolvation gas flow, 150 L/h cone gas, 150 °C source temperature, 5.0 µL/min sample flow and multiple reaction monitoring acquisition mode (MRM).

# **Additional tables**

| **Table S2 Serum levels of 25 hydroxyvitamin D2 (25(OH)D2), 25 hydroxyvitamin D3 (25(OH)D3) and total vitamin D in ST-segment elevation myocardial infarction (STEMI) coronary artery disease (CAD), non-ST-segment elevation myocardial infarction (NSTEMI) CAD and control group** | | | | |
| --- | --- | --- | --- | --- |
|  | **CAD STEMI**  (n=96) | **CAD NSTEMI** (n=22) | **Control**  (n=70) | *p*  *(ANOVA)* |
| **25 hydroxyvit D2** /nmol/L, *mean ± SD* | 3.20 ± 3.78 | 1.89 ± 3.34 | 1.58 ± 3.02 | 0.010 |
| **25 hydroxyvit D3** /nmol/L, *mean ± SD* | 55.51 ± 29.48 | 48.25 ± 24.85 | 60.76 ± 25.73 | 0.157 |
| **Total vit. D** /nmol/L, *mean ± SD* | 58.71 ± 29.40 | 50.15 ± 24.48 | 62.34 ± 25.88 | 0.195 |

| **Table S3 Allele frequency of VDR (rs2228570, rs1544410, rs731236) polymorphisms in coronary artery disease (CAD) group in comparison with control group.** | | | | | | | |
| --- | --- | --- | --- | --- | --- | --- | --- |
| **VDR (rs2228570)** | | | | | | | |
|  | **Allele A (%)** | **Allele G (%)** | **Total** | **P *(χ^2^ test)*** | **OR** | **-95% CL** | **+95% CL** |
| CAD | 126 (41) | 184 (59) | 310 | 0.71 | 1.07 | 0.75 | 1.53 |
| Control | 88 (42) | 120 (57) | 208 |  |  |  |  |
| **VDR (rs1544410)** | | | | | | | |
|  | **Allele C (%)** | **Allele T (%)** | **Total** | **P *(χ^2^ test)*** | **OR** | **-95% CL** | **+95% CL** |
| CAD | 204 (66) | 106 (34) | 310 | 0.92 | 1.00 | 0.69 | 1.45 |
| Control | 137 (66) | 71 (34) | 208 |  |  |  |  |
| **VDR (rs731236)** | | | | | | | |
|  | **Allele A (%)** | **Allele G (%)** | **Total** | **P *(χ^2^ test)*** | **OR** | **-95% CL** | **+95% CL** |
| CAD | 209 (67) | 101 (33) | 310 | 0.66 | 1.09 | 0.75 | 1.60 |
| Control | 144 (69) | 64 (31) | 208 |  |  |  |  |

| **Table S4. Genotype frequencies of vitamin D polymorphisms VDR (rs2228570, rs1544410, rs731236) in STEMI CAD group in comparison with control.** | | | | | |
| --- | --- | --- | --- | --- | --- |
|  | **STEMI CAD** | | **Control** | | *p*  *(χ^2^ test)* |
|  | *N* | *%* | *N* | *%* |  |
| **VDR (rs2228570)** |  |  |  |  |  |
| A/A | 17 | 13.7% | 18 | 17.3% | 0.710 |
| A/G | 67 | 54.0% | 52 | 50.0% |  |
| G/G | 40 | 32.3% | 34 | 32.7% |  |
| Total | 124 | 100.0% | 104 | 100.0% |  |
| **VDR (1544410)** |  |  |  |  |  |
| C/C | 54 | 43.5% | 39 | 37.5% | 0.017 |
| C/T | 51 | 41.1% | 59 | 56.7% |  |
| T/T | 19 | 15.3% | 6 | 5.8% |  |
| Total | 124 | 100.0% | 104 | 100.0% |  |
| **VDR (rs731236)** |  |  |  |  |  |
| A/A | 58 | 46.8% | 45 | 43.3% | 0.020 |
| A/G | 48 | 38.7% | 54 | 51.9% |  |
| G/G | 18 | 14.5% | 5 | 4.8% |  |
| Total | 124 | 100.0% | 104 | 100.0% |  |

| **Table S5. Inheritance models for BsmI VDR (rs1544410) polymorphism in STEMI CAD and control groups.** | | | | | | | |
| --- | --- | --- | --- | --- | --- | --- | --- |
| **VDR (1544410)** MODEL | | STEMI CAD | Control | p | OR | -95% CL | +95% CL |
| CODOMINANT MODEL 1 C/C vs T/C | C/C | 54 | 39 | 0.097 | 1.60 | 0.92 | 2.79 |
|  | T/C | 51 | 59 |  |  |  |  |
| CODOMINANT MODEL 2 C/C vs T/T | C/C | 54 | 39 | 0.103 | 0.44 | 0.16 | 1.20 |
|  | T/T | 19 | 6 |  |  |  |  |
| DOMINANTN MODEL C/C vs T/C+ T/T | C/C | 54 | 39 | 0.356 | 1.29 | 0.76 | 2.19 |
|  | T/C+T/T | 70 | 65 |  |  |  |  |
| RECESIVE MODEL  C/C+T/C vs T/T | C/C+T/C | 105 | 98 | 0.022 | 0.34 | 0.13 | 0.88 |
|  | T/T | 19 | 6 |  |  |  |  |
| OVERDOMINANT MODEL C/C+T/T vs T/C | C/C+T/T | 73 | 45 | 0.019 | 1.88 | 1.11 | 3.18 |
|  | T/C | 51 | 59 |  |  |  |  |
| ADITIVE MODEL  C/C vs 2T/T+T/C | C/C | 54 | 39 | 0.706 | 1.11 | 0.66 | 1.85 |
|  | 2T/T+T/C | 89 | 71 |  |  |  |  |

| **Table S6. Inheritance models for Taq1 VDR (rs731236) polymorphism in STEMI CAD and control groups.** | | | | | | | |
| --- | --- | --- | --- | --- | --- | --- | --- |
| **VDR (rs731236)** MODEL | | STEMI CAD | Control | p | OR | - 95% CL | +95% CL |
| CODOMINANT MODEL 1 A/A vs A/G | A/A | 58 | 45 | 0.186 | 1.45 | 0.84 | 2.51 |
|  | A/G | 48 | 54 |  |  |  |  |
| CODOMINANT MODEL 2 A/A vs G/G | A/A | 58 | 45 | 0.053 | 0.36 | 0.12 | 1.04 |
|  | G/G | 18 | 5 |  |  |  |  |
| DOMINANTN MODEL A/A vs A/G+G/G | A/A | 58 | 45 | 0.597 | 1.15 | 0.68 | 1.95 |
|  | A/G+G/G | 66 | 59 |  |  |  |  |
| RECESIVE MODEL  A/A+A/G vs G/G | A/A+A/G | 106 | 99 | 0.016 | 0.30 | 0.11 | 0.83 |
|  | G/G | 18 | 5 |  |  |  |  |
| OVERDOMINANT MODEL A/A+G/G vs A/G | A/A+G/G | 76 | 50 | 0.045 | 1.71 | 1.00 | 2.89 |
|  | A/G | 48 | 54 |  |  |  |  |
| ADITIVE MODEL  A/A vs 2G/G+A/G | A/A | 58 | 45 | 0.944 | 0.98 | 0.59 | 1.63 |
|  | 2G/G+A/G | 84 | 64 |  |  |  |  |

| **Table S7. Genotype frequencies of vitamin D polymorphisms VDR (rs2228570, rs1544410, rs731236) in NSTEMI CAD group in comparison with control.** | | | | | |
| --- | --- | --- | --- | --- | --- |
|  | **NSTEMI CAD** | | **Control** | | *p*  *(χ^2^ test)* |
|  | *N* | *%* | *N* | *%* |  |
| **VDR (rs2228570)** |  |  |  |  |  |
| A/A | 4 | 12.9% | 18 | 17.3% | 0.820 |
| A/G | 17 | 54.8% | 52 | 50.0% |  |
| G/G | 10 | 32.3% | 34 | 32.7% |  |
| Total | 31 | 100.0% | 104 | 100.0% |  |
| **VDR (1544410)** |  |  |  |  |  |
| C/C | 18 | 58.1% | 39 | 37.5% | 0.022 |
| C/T | 9 | 29.0% | 59 | 56.7% |  |
| T/T | 4 | 12.9% | 6 | 5.8% |  |
| Total | 31 | 100.0% | 104 | 100.0% |  |
| **VDR (rs731236)** |  |  |  |  |  |
| A/A | 18 | 58.1% | 45 | 43.3% | 0.046 |
| A/G | 9 | 29.0% | 54 | 51.9% |  |
| G/G | 4 | 12.9% | 5 | 4.8% |  |
| Total | 31 | 100.0% | 104 | 100.0% |  |

| **Table S8. Contingency table for the VDR polymorphisms (rs2228570. rs1544410. rs731236) regarding nonsmoking** | | | | | |
| --- | --- | --- | --- | --- | --- |
|  | **CAD**  (non-smoking) | | **Control**  (non-smoking) | | *p*  *(χ^2^ test)* |
|  | *N* | *%* | *N* | *%* |  |
| **VDR (rs2228570)** |  |  |  |  |  |
| A/A | 11 | 15.9% | 15 | 18.3% | 0.758 |
| A/G | 37 | 53.6% | 39 | 47.6% |  |
| G/G | 21 | 30.5% | 28 | 34.1% |  |
| Total | 69 | 100.0% | 82 | 100.0% |  |
| **VDR (1544410)** |  |  |  |  |  |
| C/C | 31 | 43.5% | 30 | 36.6% | 0.039 |
| C/T | 26 | 39.1% | 46 | 56.1% |  |
| T/T | 12 | 17.4% | 6 | 7.3% |  |
| Total | 69 | 100.0% | 82 | 100.0% |  |
| **VDR (rs731236)** |  |  |  |  |  |
| A/A | 32 | 46.4% | 35 | 42.7% | 0.079 |
| A/G | 26 | 37.7% | 42 | 51.2% |  |
| G/G | 11 | 15.9% | 5 | 6.1% |  |
| Total | 69 | 100.0% | 82 | 100.0% |  |
| **Table S9. Contingency table for the VDR polymorphisms (rs2228570. rs1544410. rs731236) regarding smoking when comparing smoking with nonsmoking in both groups.** | | | | | |
|  | **Smoking** | | **Non-smoking** | | *p*  *(χ^2^ test)* |
|  | *N* | *%* | *N* | *%* |  |
| **VDR (rs2228570)** |  |  |  |  |  |
| A/A | 13 | 12.0% | 26 | 17.2% | 0.484 |
| A/G | 60 | 55.6% | 76 | 50.3% |  |
| G/G | 35 | 32.4% | 49 | 32.5% |  |
| Total | 108 | 100.0% | 151 | 100.0% |  |
| **VDR (1544410)** |  |  |  |  |  |
| C/C | 50 | 46.3% | 61 | 39.7% | 0.631 |
| C/T | 47 | 43.5% | 72 | 48.3% |  |
| T/T | 11 | 10.2% | 18 | 12.0% |  |
| Total | 108 | 100.0% | 151 | 100.0% |  |
| **VDR (rs731236)** |  |  |  |  |  |
| .A/A | 54 | 50.0% | 67 | 44.4% | 0.658 |
| A/G | 43 | 39.8% | 68 | 45.0% |  |
| G/G | 11 | 10.2% | 16 | 10.6% |  |
| Total | 108 | 100.0% | 151 | 100.0% |  |

| **Table S10. Contingency table for the VDR polymorphisms (rs2228570. rs1544410. rs731236) for the male gender.** | | | | | |
| --- | --- | --- | --- | --- | --- |
|  | **CAD**  (male) | | **Control**  (male) | | *p*  *(χ^2^ test)* |
|  | *N* | *%* | *N* | *%* |  |
| **VDR (rs2228570)** |  |  |  |  |  |
| A/A | 15 | 11.8% | 9 | 13.2% | 0.904 |
| A/G | 73 | 57.5% | 40 | 58.8% |  |
| G/G | 39 | 30.7% | 19 | 28.0% |  |
| Total | 127 | 100.0% | 68 | 100.0% |  |
| **VDR (1544410)** |  |  |  |  |  |
| C/C | 56 | 44.1% | 27 | 39.7% | 0.058 |
| C/T | 51 | 40.2% | 37 | 54.4% |  |
| T/T | 20 | 15.7% | 4 | 5.9% |  |
| Total | 127 | 100.0% | 68 | 100.0% |  |
| **VDR (rs731236)** |  |  |  |  |  |
| A/A | 58 | 45.7% | 31 | 45.6% | 0.063 |
| A/G | 50 | 39.4% | 34 | 50% |  |
| G/G | 19 | 14.9% | 3 | 4.4% |  |
| Total | 127 | 100.0% | 68 | 100.0% |  |
| **Table S11. Contingency table for the VDR polymorphisms (rs2228570. rs1544410. rs731236) for the female gender.** | | | | | |
|  | **CAD**  (female) | | **Control**  (female) | | *p*  *(χ^2^ test)* |
|  | *N* | *%* | *N* | *%* |  |
| **VDR (rs2228570)** |  |  |  |  |  |
| A/A | 6 | 21.4% | 9 | 25.0% | 0.876 |
| A/G | 11 | 39.3% | 12 | 33.3% |  |
| G/G | 11 | 39.3% | 15 | 41.7% |  |
| Total | 28 | 100.0% | 36 | 100.0% |  |
| **VDR (1544410)** |  |  |  |  |  |
| C/C | 16 | 57.1% | 12 | 33.3% | 0.070 |
| C/T | 9 | 32.1% | 22 | 61.1% |  |
| T/T | 3 | 10.7% | 2 | 5.6% |  |
| Total | 28 | 100.0% | 36 | 100.0% |  |
| **VDR (rs731236)** |  |  |  |  |  |
| A/A | 18 | 64.3% | 14 | 38.9% | 0.050 |
| A/G | 7 | 25.0% | 20 | 55.6% |  |
| G/G | 3 | 10.7% | 2 | 5.5% |  |
| Total | 28 | 100.0% | 36 | 100.0% |  |
| **Table S12. Contingency table for the VDR polymorphisms (rs2228570. rs1544410. rs731236) regarding the genders.** | | | | | |
|  | **Male** | | **Female** | | *p*  *(χ^2^ test)* |
|  | *N* | *%* | *N* | *%* |  |
| **VDR (rs2228570)** |  |  |  |  |  |
| A/A | 24 | 12.3% | 15 | 23.4% | 0.006 |
| A/G | 113 | 58.0% | 23 | 35.9% |  |
| G/G | 58 | 29.7% | 26 | 40.7% |  |
| Total | 195 | 100.0% | 64 | 100.0% |  |
| **VDR (1544410)** |  |  |  |  |  |
| C/C | 83 | 42.6% | 28 | 43.8% | 0.606 |
| C/T | 88 | 45.1% | 31 | 48.4% |  |
| T/T | 24 | 12.3% | 5 | 7.8% |  |
| Total | 195 | 100.0% | 64 | 100.0% |  |
| **VDR (rs731236)** |  |  |  |  |  |
| A/A | 89 | 45.6% | 32 | 50.0% | 0.683 |
| A/G | 84 | 43.1% | 27 | 42.2% |  |
| G/G | 22 | 11.3% | 5 | 7.8% |  |
| Total | 195 | 100.0% | 64 | 100.0% |  |

**Table S13. Chronic drug treatment regimen in CAD patients and control cohort. A significant difference is observed between groups in chronic medication therapy, except for calcium channel blockers, that is due to optimal medication therapy according to the guidelines for CAD treatment.**

|  | **CAD** (n=155) | | **Control** (n=104) | | *p*  *(χ^2^ test)* |
| --- | --- | --- | --- | --- | --- |
|  | *N* | *%* | *N* | *%* |  |
| **BB** | 143 | 92.3% | 21 | 20.0% | <0.001 |
| **ACEi** | 118 | 76.1% | 13 | 12.4% | <0.001 |
| **ARB** | 19 | 12.3% | 3 | 2.9% | 0.011* |
| **ASA** | 151 | 97.4% | 6 | 5.7% | <0.001 |
| **P2Y12** | 146 | 94.2% | 0 | 0.0% | <0.001* |
| **Statins** | 151 | 97.4% | 12 | 11.4% | <0.001 |
| **CCB** | 17 | 10.9% | 8 | 7.6% | 0.382 |
| **Diuretics** | 41 | 26.5% | 6 | 5.7% | <0.001* |
| **Hypoglycemic drugs** | 33 | 21.3% | 4 | 3.8% | <0.001* |
| *^*^ Fisher exact test*  *BB- beta blockers. ACEi - angiotensin-converting enzyme inhibitors. ARB - angiotensin receptor blockers. ASA - Acetylsalicylic acid. P2y12-inhibitros. CCB- calcium channel blockers* | | | | | |
